# Exploring the Impact of Controlled Ovarian Stimulation and Non-Invasive Oocyte Assessment in ART Treatments

**DOI:** 10.3390/life13101989

**Published:** 2023-09-29

**Authors:** Romualdo Sciorio, Federica Cariati, Steven Fleming, Carlo Alviggi

**Affiliations:** 1 Fertility Medicine and Gynaecological Endocrinology Unit, Department Woman-Mother-Child, Lausanne University Hospital, CHUV, 1011 Lausanne, Switzerland; 2 Department of Public Health, University of Naples Federico II, Via Pansini 5, 80131 Napoli, Italy; cariati@ceinge.unina.it; 3 Discipline of Anatomy & Histology, School of Medical Sciences, University of Sydney, Sydney, NSW 2006, Australia; blueyfleming@gmail.com; 4 Fertility Unit, Maternal-Child Department, AOU Policlinico Federico II, 80131 Naples, Italy; carlo.alviggi@unina.it; 5 Department of Neuroscience, Reproductive Sciences and Odontostomatology, University of Naples Federico II, 80131 Naples, Italy; 6 Endocrinology and Experimental Oncology Institute (IEOS), National Research Council, 80131 Naples, Italy

**Keywords:** assisted reproductive technology, noninvasive assessment, oocyte morphology and quality, oocyte biomechanical features, healthy offspring

## Abstract

Invasive and noninvasive features are normally applied to select developmentally competent oocytes and embryos that can increase the take-home baby rates in assisted reproductive technology. The noninvasive approach mainly applied to determine oocyte and embryo competence has been, since the early days of IVF, the morphological evaluation of the mature cumulus-oocyte complex at the time of pickup, first polar body, zona pellucida thickness, perivitelline space and cytoplasm appearance. Morphological evaluation of oocyte quality is one of the options used to predict successful fertilization, early embryo development, uterine implantation and the capacity of an embryo to generate a healthy pregnancy to term. Thus, this paper aims to provide an analytical revision of the current literature relating to the correlation between ovarian stimulation procedures and oocyte/embryo quality. In detail, several aspects of oocyte quality such as morphological features, oocyte competence and its surrounding environment will be discussed. In addition, the main noninvasive features as well as novel approaches to biomechanical parameters of oocytes that might be correlated with the competence of embryos to produce a healthy pregnancy and live birth will be illustrated.

## 1. Introduction

Over the last decades, assisted reproduction technology (ART) has notably changed and is currently responsible for the birth of about nine million children [1]. Oocyte morphological assessment is an important step performed daily in routine ART procedures. However, oocyte quality is probably one of the more important limiting factors in female fertility, playing a critical role in fertilization and later during early embryo development [2,3]. Indeed, the embryo is the result of the union between the oocyte and the spermatozoon. Certainly, IVF is an aggregate of several procedures and one of these, probably the most critical, is ovarian stimulation (OS) [4]. However, during the ART cycle, the situation is more complex: in vivo, each month only one oocyte will ovulate, and its maturation takes place at the conclusion of a long period of follicle growth and selection. Clinically, the use of an OS protocol practically abolishes the natural selection of follicles and permits the maturation of oocytes that otherwise would never grow within a pool of follicles. This may be also associated with compromised oocyte and embryo competence, and could eventually be responsible for fertilization failure, embryo aneuploidy and implantation failure [5,6]. Indeed, successful fertilization is a more intricate process, which relies not only on sperm penetration but on several factors associated with oocyte quality. Therefore, the concern is that following OS, probably a good number of collected oocytes are of average quality, and thus, even with excellent laboratory conditions, they are probably designed to generate an embryo with low implantation potential, incapable of establishing a normal pregnancy to term [6,7].

However, during in vitro culture, the embryology laboratory is like the main actor, and it is extremely important that its performance is strictly correlated with embryo development and viability. The physical and chemical conditions used to culture embryos should be kept under constant surveillance, and they need to be always close to the physiological values; this is preeminent to support embryo development and guarantee its optimal implantation potential. As reported by several authors, there are concerns that suboptimal culture conditions might impair embryo development and compromise their viability [5,6,7,8]. In fact, in vitro culture involves several steps that could increase embryonic stress, including the application of different culture media, the use of plastic dishes and consumables, different oxygen concentrations, temperature, pH and osmolality. All these factors may play a critical role in embryo development and viability. Therefore, constant improvement of culture techniques to minimize embryonic stress is a necessary ongoing venture. Additionally, in relation to oocyte competence, we believe that optimal oocyte maturation cannot be determined by only observing the presence of the polar body, but rather depends upon several convoluted cytoplasmic mechanisms, which cannot be visible even with the eyes of a very experienced embryologist. Those mechanisms are extremely important to generate and store proteins, carbohydrates and lipids, and also to coordinate the metabolic processes needed for fertilization and further embryo development. In particular, oocyte quality depends not only on nuclear maturation and the appropriate number of healthy mitochondria but also on the environment within the ovary, during the time of oocyte production and maturation until the ovulatory stages [8,9,10]. Definitely, oocyte quality is a result of several aspects related to morphological features, genetic status and environmental impact on embryo development, blastocyst viability and implantation [9]. In this scenario, like a huge patchwork, the embryology laboratory which tries to mimic the physiological environment of the ovary represents only a little and small role. Therefore, we aim to summarize the current literature relating to the correlation between ovarian stimulation procedures and oocyte/embryo quality. Especially, the prognostic value of morphological features of mature (MII) human oocytes on their developmental competence will be discussed.

## 2. Search Methods

The main goal of this article will be to provide an analytical and commentary review of the literature, correlating the impact of ovarian stimulation with oocyte assessment in ART, and examining whether the stimulation protocol could have an impact on oocyte competence, embryo viability and the ability to maintain a healthy pregnancy to term. Relevant studies were identified in the English-language literature using PubMed search terms related to the focus of the review, including the relevance of OS in the ART cycle and its effect on oocyte maturation, exploring both nuclear and cytoplasmatic aspects. In addition, it also briefly analyzes the process of folliculogenesis and the importance of noninvasive evaluation of oocyte competence. All relevant publications until June 2023 were critically evaluated and discussed.

## 3. Folliculogenesis

**Figure 1:**
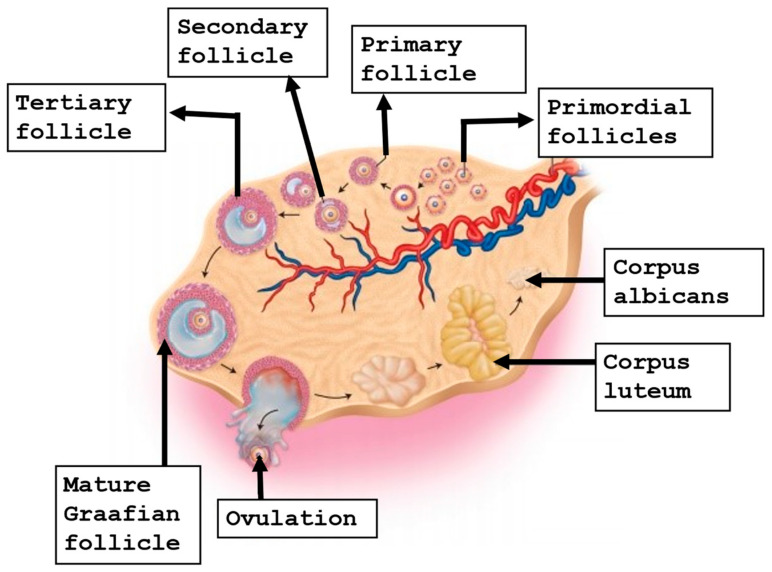
The stages of oogenesis.

Folliculogenesis is a complex interaction between the oocyte and surrounding somatic cells that starts very early around the third week of gestation: when primordial germ cells begin to grow and migrate to the gonadal ridge, where they progress into oogonia. At around week 20 of gestation, it has been estimated that oogonia reach a number of around six million [11,12]. Subsequently, the primary oocytes start meiotic division and arrest at the prophase of meiosis I, commonly called the germinal vesicle (GV) stage. They will stay at that stage until puberty, when they will be reactivated by circulating gonadotrophins [13]. At around six months of gestation, the oocyte is nestled within the primordial follicle, which at this stage is enclosed by a single layer of flattened granulosa cells. Later, some primordial follicles undergo growth and differentiation, with the conversion of the granulosa cells from flattened to cuboidal cells, to evolve into primary follicles [14,15]. Afterwards, the oocyte continues to grow, and eventually, the primary follicle becomes a secondary follicle. At that time, the layer of granulosa cells expands and develops gap junctions, and the receptors for follicle-stimulating hormone (FSH) [16]. Under the action of FSH, a small fluid-filled cavity will comprise the antrum that furnishes nutrients and mediates waste removal for the oocyte [17]. With antrum cavity formation, the follicle progresses and forms the tertiary follicle, and as the antrum continues to enlarge, it will form the preovulatory follicle [18]. The group of granulosa cells in proximity to the oocyte are called cumulus cells, while the layer of cumulus cells which are in direct contact with the oocyte are termed the corona radiata [19]. The preovulatory surge of luteinizing hormone (LH) triggers the GV-arrested oocyte to resume meiotic division to generate the mature cumulus oocyte complex (COC), which encloses an oocyte arrested at MII [20,21]. It is essential that MII oocytes contain a meiotic spindle to provide a regular chromosome alignment to avoid aneuploidies in the future embryo [22,23]. Figure 1 shows the stages of oogenesis.

## 4. Oocyte Competence in ART Cycles

Evaluation of oocyte quality can be performed using the microscope without damaging their initial developmental potential and without interfering with subsequent embryonic development. Morphological evaluation can be considered noninvasive and is normally compatible with the workflow of ART treatment. Human oocytes not only provide genetic material to the developing embryos but are also responsible for supplying energy substrates, nutrients and a mitochondrial genome. An altered expression of genetic information could be caused by defects in the DNA, protein-histones, cytoskeletal system, the DNA repair mechanism and systems that regulate gene expression and many other metabolic processes. The ability of an oocyte to complete meiotic maturation has been called “meiotic competence”, while the capacity to achieve successful embryonic development is termed “developmental competence”. Either association will contribute to “oocyte competence” [8,9,10]. It is well accepted that maternal age is the most predictive feature of oocyte competence. Unfortunately, advanced female age significantly affects pronuclear size and intra- and extra-nuclear dynamics during fertilization, dysregulates cell polarity during compaction, impairs morula formation and reduces blastocoel expansion [22,23]. However, some of the most evident benefits of better predictive assessment of oocyte competence are higher live births and lower pregnancy loss rates [24]. An additional benefit of increasing the ability to predict oocyte developmental potential would be the further adoption of single embryo transfer, which is critical to and extremely decisive in reducing the morbidity, complications, as well as the higher costs associated with multiple gestations [25]. Further, it is worth mentioning that as the ability to predict oocyte competence increases, OS procedures could be enhanced and amended in order to preference the quality over quantity of oocytes collected, making patient treatment more favorable and cost-effective globally, reducing the percentage of ovarian hyperstimulation syndrome (OHSS) [26]. Unluckily, the occurrence of OHSS remains one of the major complications seen in ART, which is a potentially life-threatening condition [27]. Currently, there are usable clinical features, as well as OS protocols, that help to reduce the occurrence of this syndrome [28]. However, over the last couple of decades, with the introduction of vitrification, we have witnessed a remarkable advancement in embryo cryopreservation, as a routine procedure and alternative to the slow-freezing method, owing to the superior success rates in terms of cryo-survival and pregnancy outcomes [29]. Therefore, the application of the freeze-all strategy can be considered as the gold standard useful in reducing the risk of OHSS [30]. The benefit of the freeze-all feature was first introduced more than 20 years ago [31]. Over the years, several authors have analyzed the efficacy of elective cryopreservation of all embryos as compared to a fresh embryo transfer in reducing the risk of OHSS [32,33,34,35]. Moreover, consistent data demonstrate that the cumulative live birth, biochemical pregnancy rate, clinical pregnancy, ongoing pregnancy and pregnancy loss are similar between fresh transfer and frozen transfer, conferring high efficiency to the strategy [33,34]. However, with the application of vitrification, a critical concern is how to cryopreserve human materials in the safest way and to avert cross-contamination. The strategies normally adopted are the open and closed procedures: the former involves direct contact between liquid nitrogen (LN_2_) and the sample, while the latter avoids that contact with LN_2_, which will also reduce eventual risk of contamination during the vitrification and storage processes. In our view, and in agreement with several authors, vitrification of human oocytes and embryos should be carried out in extremely and rigorously safe regimes, thus the closed vitrification system should be the way to go for these types of cells [36,37,38]. Finally, it is important to mention that especially for those patients at risk of OHSS, as well as all the couples undergoing ART treatments, adequate psychological support should be always offered in order to deal with various infertility pathway complications [39].

## 5. The Relevance of OS in ART

OS requires the use of exogenous gonadotrophins to stimulate the woman’s ovaries to generate multiple oocytes, which are retrieved transvaginally [4]. The process aims to overcome the selection of a single dominant follicle and to retrieve multiple oocytes in a single stimulated cycle [4,40]. In order to improve the efficacy and efficiency of OS, several studies have been performed to better understand the molecular action of gonadotrophins. FSH and LH mediate steroidogenesis, apoptotic events and maturation of the dominant follicle through the specific G-protein-coupled receptors, FSH receptor (FSHR) and LH/Chorionic Gonadotrophin receptor (LHCGR) [41]. Consistent with gonadotrophins’ functions, the expression of receptors changes dynamically throughout folliculogenesis, especially during the increase in follicular diameter, when the expression of LHCGR increases, while FSHR-1 decreases [42]. In particular, the FSHRs are mainly expressed by granulosa cells of the developing ovarian follicle; instead, LHCGRs are primarily expressed by theca cells of the early antral follicle, the mural granulosa cells of the periovulatory Graafian follicle and the luteal cells of the corpus luteum [43]. According to the two-cell–two-gonadotrophin theory, cooperative interactions of FSH and LH have been demonstrated in vivo. Data were derived from hypophysectomized rodents treated with high doses of FSH that were able to boost the final stages of follicular maturation and trigger ovulation thanks to cooperation with a residual population of the LHCGR. This was later confirmed in LHCGR-knockout rodents that did not show progression of folliculogenesis and induction of ovulation when treated with FSH [44,45]. It has been clearly demonstrated that, during the gonadotrophin-dependent phase, FSH is able to act via the heterodimer of the receptors (FSHR-LHCGR) even when the level of LH is low [43]. Gonadotrophins exert their role through life-and-death molecular signals [41,46]. Especially during the intermediate–late follicular phase, stimulation of the LHCGR drives the dominant follicle, activating ERK1/2- and AKT-dependent proliferative and anti-apoptotic signals triggered by the action of LH [41]. Clinical studies have demonstrated that LH activity stimulates the growth and supports the maturation of larger (about 10–14 mm) follicles, while simultaneously being able to selectively reduce the occurrence of small preovulatory follicles [47]. There is clinical evidence that, following FSH priming, LH alone is capable of effectively completing follicular maturation. Nevertheless, the optimal amount of LH activity supplementation needed for OS is still unclear [47]. One of the main issues encountered by embryologists is to recognize the quality of the oocytes obtained from follicles independently of the apparent evidence of the first polar body (PBI). Indeed, oocyte competence, as known, is not always homogeneous in a pool of oocytes retrieved after OS [48]. During the process of growing, the follicle enlarges, and the oocyte diameter increases to reach the dimension of about 100 µm (approximately 4-fold increase) over a period of 8 weeks; thus, a 64-fold increase in volume occurs, resulting in enhanced cytoplasmic activity [48]. Maternal transcripts and proteins are stored during the growth of developing oocytes and are essential to functionally regulate a broad range of nuclear condensates, including nuclear speckles and nucleoli [49]. As a result, it is difficult to correlate OS treatment with oocyte quality. For this reason, the impact of the gonadotrophins used for OS on oocyte competence remains controversial. In a recent study, Vaiarelli and colleagues reported that MII oocyte competence is unlinked to the gonadotrophins used for OS [50]. More specifically, at least in advanced maternal age, the OS regimen does not seem to affect the euploid blastocyst rate per MII oocyte. Conversely, two recent meta-analyses seem to support the hypothesis that supplementation with recombinant human LH (r-hLH) improves the outcome of IVF independently of oocyte yield [50,51,52]. In particular, LH supplementation significantly increases the clinical pregnancy rate in women between 35 and 40 years of age [52]. This effect seems to disappear when trials performed in women above 40 years of age are analyzed. In addition, post hoc logistic regression analysis from a large randomized controlled trial (RCT) in patients with poor ovarian response (POR) describes a lower incidence of total pregnancy outcome failure in women treated with r-hFSH/r-hLH compared with those receiving only r-hFSH [53]. Taken together, the information deriving from in vivo experimental models and clinical trials seems to reinforce the concept that LH is crucial in supporting late phases of oocyte maturation, which in turn improves embryo competence. During OS, exogenous LH might play a relevant role in counteracting age-dependent decrease in oocyte quality. This effect is more evident in women between 35 and 40 years of age [51] and apparently not related to the rate of aneuploidies [50], suggesting the involvement of cytoplasmic-dependent mechanisms.

### 5.1. Mild or Moderate Ovarian Stimulation

Currently, there is not enough scientific proof to approve the hypothesis that gonadotrophin dosage influences oocyte competence. Indeed, clinical data failed to prove that mild stimulation is associated with better gamete quality, even in women with low prognosis [54]. Conversely, current data support the idea that oocyte yield is independent of the age-related rate of aneuploidies, meaning that the higher the number of MII oocytes, the greater the absolute number of euploid embryos. In light of this evidence, it has become essential to collect a necessary number of oocytes to ensure at least one good-quality/euploid embryo. This strategy appears more effective when a conventional protocol is applied. Moreover, in poor-responder patients, the accumulation strategy could represent a valuable approach [54].

### 5.2. Full Stimulation and Risk of OHSS

OHSS is one of the main complications related to OS, due to human chorionic gonadotrophin (hCG) used to induce final oocyte maturation and/or endogenous hCG produced by implanted embryos [55]. However, as described earlier, many strategies have been used to reduce or avoid OHSS. Specifically, OS with gonadotrophin-releasing hormone (GnRH) agonist triggering to avoid exposure to exogenous hCG, vitrification of all oocytes and/or embryos, and single embryo transfer after thawing in a receptive endometrium represent the main strategies to dramatically reduce the risk and to obtain an OHSS-free clinic [56].

### 5.3. Long-Acting Recombinant FSH

Traditionally, OS requires daily injections of FSH from cycle day 2 to induce the growth of multiple follicles within the ovary. However, a recombinant long-acting FSH, known as corifollitropin alfa or FSH-CTP, allows a more patient-friendly weekly subcutaneous administration while maintaining systemic levels of FSH necessary for multi-follicular growth [57]. A Cochrane systematic review of six RCTs, including 3753 patients aged 18–41, concluded that there was no significant difference in clinical outcomes or adverse events between those administered a medium dose (150–180 µg) of FSH-CTP and those receiving daily injections of FSH [58]. The ENGAGE trial, which compared FSH-CTP with recombinant FSH over the first seven days of OS in 1506 patients with a mean age of 31.5 years, found that there was a significantly higher number of oocytes retrieved (13.7 ± 8.2 vs. 12.5 ± 6.7) following the use of FSH-CTP [59]. Similarly, the ENSURE trial also reported a significantly higher number of oocytes (13.3 ± 7.3 vs. 10.6 ± 5.9; *p* < 0.001) following OS using FSH-CTP [60]. However, The TRUST and the PURSUE trials [60,61,62] reported that there was no significant difference in the number of oocytes retrieved, and these findings were supported by an individual data meta-analysis of three RCTs [63]. In potential poor responders, significantly higher numbers of oocytes (4.8 ± 2.1 vs. 3.6 ± 2.2) have been observed following OS with FSH-CTP [64]. Using the mouse model, it has been recently suggested that long-acting FSH enhances follicle development and supports oocyte maturation and embryonic developmental potential in vitro [65].

### 5.4. OS with Adjuvant Treatment

Following conventional OS using GnRH analogues with gonadotrophins, poor responders are characterized as having low numbers of oocytes retrieved. Since POR may be unpredictable, the POSEIDON criteria were developed to better classify its definition [4]. To mitigate recurrent POR, various adjuvant or complementary treatments have been combined with OS, including androgens, androgen-modulating agents, ovarian steroids, growth hormone (GH) and the coenzyme Q10 [66]. Depending upon their mode of action, the putative potential benefits of various adjuvant treatments include enhanced oocyte maturation and embryo quality, though an impact upon endometrial receptivity is also a possibility. However, the use of adjuvant therapies remains controversial due to the variability in OS and the classification of POR in different studies and has not been previously recommended by ESHRE [67]. A network meta-analysis of 17 RCTs, including 1680 women, showed that the adjuvants that resulted in the highest numbers of oocytes retrieved were hCG, oestradiol and GH [66]. Furthermore, in a meta-analysis of 19 RCTs, including 2677 women, dehydroepiandrosterone (DHEA) and coenzyme Q10 were found to significantly enhance the clinical pregnancy rate [66]. Generally, GH seems to be the adjuvant of choice for POR since it maximizes the number of oocytes and embryos while significantly reducing the gonadotrophins required for OS. This may be relevant since there is some evidence that exposure to higher levels of FSH could be detrimental to oocyte and embryo quality and euploidy [66,67,68,69]. Recently, using the aged mouse model, in vivo administration of GH has been shown to restore spindle assembly and reduce aneuploidy rates within oocytes [70]. It has been suggested that GH may achieve such benefits by stimulating intra-ovarian insulin-like growth factor-I [71] and by enhancing the action of gonadotrophins on granulosa cells [72].

### 5.5. Double Stimulation (DuoStim): Is It Risky?

Tocci and co-authors, in 2022, proposed mechanisms by which the DuoStim approach could be unsafe [73]. Based on studies performed in vitro or using animal models, the authors argued that the second stimulation is potentially able to trigger ovarian stem cell differentiation through a persistent action of FSH on functional FSHRs expressed in human pre-antral ovarian cells [73]. In addition, they mentioned a list of FSH-dependent intracellular oncogenic signaling pathways supposing a tumorigenic activity of gonadotrophins. According to Casarini and colleagues [74], these hypotheses are not consistent with currently available data. More specifically, no evidence in humans demonstrates either the presence of ovarian stem cells in the ovary or the existence of FSHRs in pre-antral ovarian follicles. In addition, an eventual differentiation from ovarian stem cells to an oocyte embedded into a primary follicle takes a duration of time that does not fit within the DuoStim timespan. Indeed, the FSH downregulates FSHR mRNAs, which are replaced by LHCGR transcripts, and data do not exist about their persistence. Finally, an increase in cancer risk in IVF patients is well appreciated to be mild compared to the general population [73].

## 6. Oocyte Nuclear Maturation

**Figure 2:**
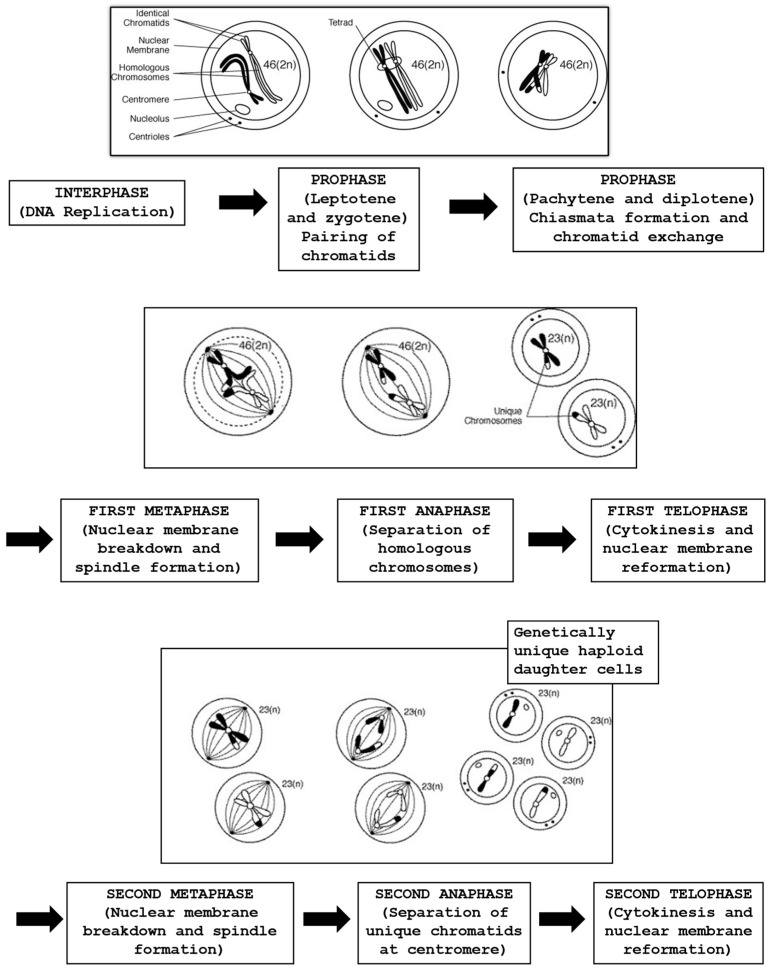
The process of meiosis with the formation of haploid gametes.

In modern ART cycles involving ICSI, prior to injection, the oocyte needs to be treated with a specific enzyme to eliminate the cells surrounding the oocyte, permitting the embryologist to visually perform sperm injection into the MII oocyte cytoplasm. The process begins with the oocyte’s exposure to the hyaluronidase enzyme, succeeded by mechanical force applied using a 130–135 µm glass or plastic pipette. This allows for clearly determining the stage of oocyte nuclear maturation (GV stage, metaphase I (MI), anaphase I, telophase I or MII)). The evaluation by light microscopy of the PBI in the perivitelline space (PVS) is considered a marker of nuclear maturation. Oocytes with clear extrusion of the PBI are at MII, with the chromatin aligned on the equatorial plate of the meiosis II metaphase spindle [74,75]. An accurate oocyte maturation at MII might be confirmed by the identification of the meiotic spindle (MS), which can usually be localized adjacent to the PBI, and its function is essential for correct chromosome segregation, whereas its dysfunction can induce embryo aneuploidies [76]. Several studies have investigated the importance of the MS in human oocytes, and its presence has been correlated with fertilization rates and pregnancy outcomes with contradictory results. Some authors reported that oocytes with an MS showed significantly higher fertilization, pregnancy and implantation rates [77,78], whereas others [79] did not find a significant difference. However, it needs to be considered that the daily routine work in the embryology laboratory might affect the architecture and the cytoskeleton of the oocyte, damaging the MS and eventually being responsible for lower fertilization rates [80]. Generally, with OS it is predicted that about 80–85% of the oocytes are at the MII stage, with a clear presence of the PBI, with around 5–10% at the GV stage, and another 5–10% of the oocytes with absence of both PBI and GV are classified as being at MI. These oocytes have gone through GV breakdown but have not fully completed meiosis I and are still between MI and MII, where the chromosomes are aligned on the metaphase plate in preparation for finishing the first meiotic division [81]. Figure 2 depicts different stages of the meiosis process.

### 6.1. From Birth to Puberty: Oocyte Chromatin Segregation and Resumption of Meiosis

At birth, the primordial follicle containing the oocyte is quiescent at the prophase of the first meiotic division. The oocyte chromosomes are dispersed and transcriptionally active to operate the basic level of activities. During follicle evolution, the oocyte sustains a growth phase, in which the oocyte achieves full size and is ready to ovulate. This stage is animated by intense RNA transcription; therefore, oocyte chromatin needs to be dispersed to permit interaction with the transcriptional machinery. Once growth is completed and the oocyte reaches the capability to restart meiosis (meiotic competence), it will undergo a considerable DNA condensation process; thus, chromatin compaction is transcriptionally inactive in preparation for meiotic resumption [82]. The chemical compound cyclic adenosine monophosphate (cAMP) plays an essential role in the regulation of meiotic arrest before ovulation. To appreciate more what might represent biomarkers of proper oocyte developmental competence, we should look carefully at the process of meiosis, which starts with the replication of the genetic material during the S-phase. This is then followed by two successive chromosome segregations, which results in a haploid chromosome constitution [82,83]. The meiotic process allows the reduction of the chromosome numbers to a haploid set and comprises the recombination of new genetic combinations in the offspring due to an exchange of genetic material between paternal and maternal homologues. The spindle apparatus is a cytoskeletal structure that is actively involved in the separation of homologous chromosomes during meiosis I and sister chromatids during meiosis II to produce haploid gametes with half the number of chromosomes of the parent cell [83,84,85]. The spindle fibers are formed by filaments called microtubules, which are dynamic structures that can disassemble and reassemble, since they are made of heterodimers of alpha and beta tubulin in association with microtubule-associated proteins (MAPS) [78,81,85]. Most importantly, oocyte spindle stability and function might be altered by suboptimal conditions in the embryology laboratory. Oocyte MS stability can be influenced by non-physiologic pH and temperature. Thus, the human MS begins to depolymerize at a temperature of 33 °C [86] and continues to depolymerize as temperatures drop, and it has been reported that only about 10 min of exposure to non-physiologic pH is sufficient to induce spindle disassembly [85]. Those relevant studies corroborate that it is critical to carefully monitor IVF laboratory conditions and avoid fluctuations in temperature and pH. Consequently, MS dysfunction during specific developmental times of active chromatin segregation may be associated with abnormal chromosome segregation and therefore directly responsible for aneuploidies in the oocytes and later in embryo development. Unfortunately, it has been reported that maternal age might negatively influence some essential spindle association checkpoints, which explains the increased rate of MS alterations and aneuploidies in embryos produced in patients in advanced maternal age [87]. A study published by Capalbo and colleagues reported that in women over age 40, almost 80% of gametes display abnormal spindles and chromosome misalignment, compared to only 20% in younger patients aged 25 or under [88]. Generally, as mentioned earlier, live birth rates remain suboptimal in women with advanced maternal age. The main cause of this poor outcome is probably high rates of embryonic aneuploidy. Thus, preimplantation genetic testing for aneuploidies (PGT-A) on embryos and later noninvasive prenatal testing (NIPT) or invasive prenatal diagnostic analysis are strongly recommended in order to improve ART outcomes [89,90].

### 6.2. Cytoplasmic Maturation

Oocyte nuclear maturity alone is not sufficient for determining oocyte and future embryo quality. Oocyte competency is not only reliant on the nuclear and mitochondrial genome but is also susceptible to cytoplasmic maturity [91]. Female gametes also carry mitochondria, which enclose their own DNA (mt DNA), which is fully provided by the maternal germline: a mature oocyte contains more than 150,000 copies of mt DNA [92,93]. Cytoplasmic evaluation should be taken into consideration to determine ideal conditions for subsequent fertilization and embryo development. An MII oocyte should then consist of a typical clear-looking cytoplasm, a clear, smooth, and non-fragmented PBI, an adequate zona pellucida (ZP) thickness and a small PVS [6]. Regrettably, those evaluations are subjective and might diverge according to the operator’s experience, and thus it is hard to have a validated predictive value in assessing the molecular signature of oocyte cytoplasmic maturation. These molecular mechanisms and signaling in the oocyte cytoplasm are essential for the production and storage of carbohydrates, proteins, RNAs, lipids and fatty acids, successful organelle position and regulation of metabolic pathways required for oocyte maturation, competence for fertilization, and subsequent embryonic developmental capacity [6].

### 6.3. Polar Body Appearance

The PBI is located in the PVS and is typically smooth and without fragmentation. The biological and physiological relevance of PBI morphology, fragmentation or dysmorphism is currently obscure and still a subject of big debate. PBI fragmentation should not be addressed as an oocyte marker since the fragmentation may be associated with the post-ovulatory period. However, it has been proposed that a degenerated PBI might be correlated with asynchrony between nuclear and cytoplasmic maturation, probably due to the post-maturity of the oocyte [94]. According to some authors, oocytes showing a clear and intact PBI without any fragmentation have a raised capacity to generate blastocysts and higher pregnancy rates [95,96]. However, studies have been conducted with the aim of establishing the relationship between PBI morphology and ICSI outcome, but results did not show a neat link between the two characteristics [97,98]. Further, a large PBI can be considered as a feature of poor prognosis and relates to compromised embryo viability, and an increased percentage of embryos with multinucleated blastomeres and, thus, might support embryo aneuploidies [97,98,99]. Published studies seem to agree that most of the aneuploidies in early-stage human embryos are carried from meiotic errors arising during oogenesis [83,100]. Recently, it has been proposed that chemical compounds and environmental pollutants, including endocrine disruptors, are depicting a considerable warning to human reproductive health [101]. On that line, PGT-A has been encouraged with the aspect of determining euploid embryos to be replaced in IVF cycles [102]. In particular, PB biopsy, first introduced by Verlinsky and collaborators [103], represents an alternative to day-3 or day-5 biopsy. An advantage of this application is the longer time available to perform genetic testing without the need to vitrify the embryo; also, it avoids embryo manipulation, which might be critical in those countries where embryo manipulation is not allowed. However, the large disadvantage of the PB biopsy technique is that it can only discriminate maternal aneuploidies and cannot identify paternal meiotic or post-zygotic mitotic errors. Additional information on the application and results of PGT-A have been published by others [103,104,105,106,107].

## 7. The Influence of Noninvasive Evaluation of Oocyte Quality

One of the most difficult challenges for the clinical embryologist is to select from a cohort of embryos the single one to transfer, taking into consideration the restricted information available, on the embryo’s viability, using standard morphological evaluation. It is well known that a considerable proportion of morphologically defined good-quality embryos still fail to implant and generate a pregnancy to term, even following PGT-A. Surely, the goal of ART should be the delivery of a singleton healthy baby; thus, elective single embryo transfer should be necessary and routinely applied to minimize the risk and difficulties correlated with multiple gestations [108,109]. Therefore, the need to validate and adopt a noninvasive process to determine typical features of oocytes and embryos that emulate normal health or the ability to further develop into a healthy pregnancy to term is demanding. A method, to be definitely noninvasive, should be not harmful and not disruptive to the physiology of the oocyte, or its capacity to be fertilized and to develop further to implant and result in a healthy baby. For this purpose, historically, morphological microscopic assessment has been applied to evaluate an embryo’s viability. The observation at light microscopy of an oocyte’s cytoplasm has been the subject of many published trials that try to determine its association with fertilization and pregnancy outcomes. Some studies have investigated the oocyte cytoplasm and have defined the atypical aspect as “dark cytoplasm” [110], while others found a “dark granular appearance of the cytoplasm” [111], “dispersed cytoplasmic granularity” [112] or “dark cytoplasm with granulation” [113]. Thus, some authors carefully examined these cytoplasmic characteristics and attempted to establish whether they have an impact on pregnancy outcomes. It was reported that dark cytoplasm was not a predictive factor; thus, it correlated neither with the fertilization rate nor with the embryo quality [110,111,112,113], while other authors showed that embryo quality was compromised when embryos developed from oocytes with dark cytoplasm [114]. A trial performed by Wilding and collaborators showed that cytoplasmic granulation was associated with higher fertilization rates compared to oocytes without any granularity [115]. Therefore, the debate on dark and cytoplasmic granularity is still active; however, it is worth mentioning that these evaluations are very subjective and might have considerable discrepancies and variations between embryologists and laboratories. Due to the progress in laboratory technologies, more objective assessments based on morphokinetics and morphometrics have been introduced to monitor fertilization such as PB2 extrusion, pronuclear formation and embryo development at both cleavage and blastocyst stages [116,117]. The application of time-lapse systems could be considered as a valuable approach to reduce the inter-variation between operators and provides a better evaluation of fertilization, cyto-dynamics, cell division, morula and blastocyst formation, which can be adopted as a noninvasive assessment. In addition, continuous evaluation of embryo development allows to discriminate those abnormal phenotypes that otherwise would be less frequently observed during standard culture, including abnormal cleavage, reverse cleavage, multinucleation and blastocyst collapse events, just to mention some [116,117,118,119,120,121]. Furthermore, the application of novel generations of microscopies, such as polarized microscopy, hyperspectral microscopy and Raman microspectroscopy (RM), will be valuable to assess not only the metabolic state of the embryo but also to understand more about early embryo assessment and implantation potential [100]. Montag and colleagues have revisited the use of polarized optics to assess human oocytes [122]. One of the principal advantages of this technology is that it is noninvasive imaging, it can be performed in real time and on living cells. It can detect the intracellular organelles of gametes and embryos, including spindle visualization [123] or the organization of ZP around the oocyte [124,125]. RM is a combination of Raman spectroscopy and confocal microscopy and can be used to identify interactions between light and live matter. The photon scattering generates unique spectra that can be used to detect molecules and their molecular bonds in living cells [126,127,128,129]. These novel technologies are in continuous evolution and have distinguished themselves for having very low phototoxicity, making them ideally suited for studies of development and cellular dynamics [128]. Studies using RM were able to identify patterns of intracellular lipids and areas of high protein content and describe significant differences in lipid and protein components, as well as mitochondrial identification [129,130,131]. Such preliminary studies, however, need to be further investigated, to examine the intra-structure function and organization of human embryos, to obtain biomarker data on oocyte competencies and to advise on the selection of embryos to transfer in ART.

## 8. Further Concerns and Conclusions

This manuscript has highlighted the complexity of human oocyte quality and early embryo development. Taking into consideration the revision of the literature discussed regarding the impact of morphological features and the surrounding environment on oocyte quality and competence, some conclusions have been drawn. The integration of biochemistry and other biomedical engineering technologies into the basic studies of oocyte biology and physiology represents the future of noninvasive evaluation of oocyte competence. However, these technologies are not easy to implement, but progress is being made. It will be important to support and promote research in multiple subjects correlated together in order to create a truly evidence-based science that will serve as a decent biomarker of oocyte quality and embryo developmental competence, associating with the developing technologies and platforms that are truly noninvasive and compatible with daily clinical laboratory activities in the area of ART. OS and culture conditions in the embryology laboratory are uniformly critical in this journey toward the production of viable oocytes and embryos capable of generating healthy pregnancies to term. Embryo culture in physiological conditions and using the application of time-lapse assessment, as well as some novel microscopy technologies, such as RM described above, may help the embryologist team to be able to select the single embryo to be transferred and to increase the likelihood of optimal pregnancy and reduce to the minimum the incidence of multiple gestations.

## Data Availability

No data are available.
